# Failure in Identity Building as the Main Challenge of Infertility: A Qualitative Study

**Published:** 2020

**Authors:** Shaghayegh Alamin, Tallat Allahyari, Behzad Ghorbani, Ali Sadeghitabar, Mohammad Taghi Karami

**Affiliations:** 1- Department of Social Work, Faculty of Social Sciences, Allameh Tabataba’i University, Tehran, Iran; 2- Nanobiotechnology Research Center, Avicenna Research Institute, ACECR, Tehran, Iran; 3- Avicenna Infertility Clinic, Avicenna Research Institute, ACECR, Tehran, Iran; 4- Monoclonal Antibody Research Center, Avicenna Research Institute, ACECR, Tehran, Iran; 5- Department of Women’s Studies, Faculty of Social Sciences, Allameh Tabataba’i University, Tehran, Iran

**Keywords:** Blood connection, Counseling, Culture, Femininity, Identity, Infertility, Masculinity, Social context

## Abstract

**Background:**

The approach of considering the psychosocial consequences of infertility has become significant. Psychosocial outcomes of infertility are defined and shaped in the context of the particular social and cultural conditions. Childbearing, and raising a child are considered to be the core of “identity” in many collectivist cultures, and the status provided to individuals with children is accompanied with social acceptance and approval. In such societies, infertile people see their social identity seriously damaged. The purpose of this study was to comprehend the challenge of identity crisis of infertile people in Iran for helping to formulate support programs for policy makers.

**Methods:**

This qualitative study was conducted in 2016–2018, through semi-structured interviews conducted with 40 infertile clients of Avicenna Fertility Center. Data were analyzed by Strauss and Corbin coding paradigm.

**Results:**

The core of the phenomenon of psychosocial consequences of infertility was failure in identity building, which included the main categories of prevention from continuity and prevention of forming normative identity.

**Conclusion:**

Challenge of identity crisis based on the experience of infertile people and the social construction of infertility in their minds affects not only internal, external, personal and structural factors, but it is also a kind of identity search in individuals. Regarding this fact, providing appropriate social services and training the skills needed to rebuild identity of infertile people and their social health will be very effective.

## Introduction

Fertility is especially important in developing countries according to their social norms and cultures. Even in today’s world, with regard to changes in family values, parental experience for men and women is an undeniable matter, and is a measure to achieve personal satisfaction, social acceptance, and social identity. In fact, health and disease are largely determined by the social-context and it should be considered when to distinguish normal and abnormal conditions. It can be argued that the social structure of health and disease may be even more significant in the field of infertility as compared to other conditions. In societies where marriage and childbirth are considered very important, it is likely to see more negative effects in confronting infertility. Infertility in developing countries is defined as an unperfected body and unfulfilled human identity, and the main paradox of infertility is that it is generally more common in societies in which childbearing is particularly important for the community. According to the World Health Organization, over 80 million people are infertile in the world, and infertility rates vary between 5% and 30%. Most infertile people live in developing countries. Iran with an infertility rate of 24.9% is considered to be a country with high prevalence of infertility (1). The World Health Organization has classified the psychosocial consequences of infertility in six levels, the first two levels are psychological outcomes (1: fear, guilt, self blame; 2: marital stress) and the other levels are social outcomes of infertility (3: mild marital or social violence, social isolation, 4: severe economic deprivation, moderate to severe violence, total loss of social status; 5: violence induced suicide, starvation/disease; 6: lost dignity in death) (2).

A look at international foreign research on the consequences of infertility suggests that these studies, as well as more domestic researches on infertility, have been conducted with psychological approaches. A remarkable point in international researches is that most of these studies have been done in Asian countries such as Vietnam, Palestine, Japan (3), Kuwait (4), and Turkey (5). This finding can confirm the importance of parenting and the role of parenthood in Eastern and Third World countries, which have collectivistic and traditional cultures, and family structure and kinship are of particular importance. For example, in providing descriptive literature on infertile women (6–11), some examples of identity formation of infertile women or infertile couples from qualitative studies are negative identity, sense of worthlessness and inadequacy, feeling of lack of personal control, anger and resentment, grief and depression, anxiety and stress, lower life satisfaction, envy of another mother, loss of the dream of co- creating, the emotional roller coaster and the sense of isolation.

Many of the above studies are reminiscent of an argument that women consider infertility as a direct strike to their identity (12). Infertility in women prevents them from fulfillment of their femininity, and infertile women do not fully identify their femininity from a personal and social aspect. Having a child is essential for the development of gender identity of women and an adult in many cultures. In some societies, emphasis is placed on motherhood in shaping women’s identity more than others. A study reports that femininity and motherhood are mixed in northern Vietnam and asserts that trying to have a child is in fact an attempt to shape a “normative identity” (13). In Cameroon, infertility can be the cause of women’s poverty (14), because fertility is considered as the centerpiece of women’s identity. In developing countries, infertile women and men may have resistance to consider themselves infertile (15) because they want to prevent their identity from being damaged. The experience of treatment in infertile women can be categorized in three situations: (1) the loss of control over their personal lives leads them to treatment. The process of their treatment further loses their control (2) the loss of their physical integrity leads them to treatment and the treatment process causes more invasion of their bodies (3) the sense of losing their identity leads them to treatment and the treatment makes them feel they are not treated like the rest of people (16). A holistic approach to patient care is believed to improve health outcomes, increase patient and team satisfaction, reduce negative psychosocial reactions and help patients better come to terms with their experiences (17).

In addition, review of the literature has revealed that the socio-cultural context in which the infertile couples living affects all aspects related to infertility (18, 19). Nevertheless, studies of fertility in developing countries are in most cases done with a focus on the biomedical, moral or psychological aspects of the issue with less attention to socio-cultural context (20).

Accordingly, developing countries need to reevaluate social and health care in order to cope with the consequences of infertility in society. Given that infertility is emerging as a growing crisis in the lives of individuals and a social problem, it has become prevalent in a society with cultural and social characteristics of Iran. On the other hand, the necessity of accurate recognition of this phenomenon becomes apparent as infertility is one of the important issues in the process of demographic changes. Therefore, the main aim of the present research was to extend the professional and native knowledge of social work to the phenomenon of failure in identity building as a challenge of infertility for individuals and families in contemporary societies. The secondary aim of research was to provide guidance for social policy and formulation of supportive programs to address this issue and enrich the practical activities of social workers.

Since cross-sectional quantitative studies are still common in dealing with the psychosocial consequences of infertility, regardless of their inadequacies in sorting out cause and effect, this study was conducted qualitatively to examine the main challenge of infertility in Iran.

## Methods

The present study in terms of purpose is fundamental-practical and in terms of method is descriptive-analytical and was conducted as a qualitative study. Strauss and Corbin coding paradigm was drawn to manage and to analyze data gathered from the participants who were couples with primary infertility with no surviving children. Study setting was Avicenna Fertility Center in Tehran, where many infertile couples from different parts of country are seen annually. Sampling procedure was done using a purposeful sampling strategy and the interviews continued until data saturation. So a total of 40 participants were included. Inclusion criteria consisted of primary infertility, having no surviving child, having no adopted children and willingness to participate in the study. The characteristics of participants in the study sample with maximum variance assured the quality of the study by strengthening the validity and transferability of findings.

Next, informed consent was obtained from all participants after explaining the study and the need to obtain audio recording by main investigator. Data was collected using the semi-structured interviews by recording voices. Data collection in the interview was done by categorizing questions into five general categories. First, a general question was asked to identify the history of people (Please give a brief summary of yourself and your experience, and where did the story begin?). Then, information was gathered with five main themes: information before finding out about infertility, information about the time after infertility diagnosis, actions taken, after-action information, and current status of individuals. Data collection and analysis lasted from December 2017 to June 2018.

Data analysis was done through the systematic classification process to identify codes and themes within the content of the study. In addition, codes were extracted from the meaningful units of the participants descriptions and were classified with reference to similarities or dissimilarities, based on the relevant themes identified by the paradigm (21, 22). Several measures were taken to strengthen and distribute credibility of the data such as assuring the adequate diversity of participants in terms of socio-demographic features, increasing contact time with participants, clarifying the objectives of the study for the participants, and analyzing transcriptions immediately after the interview. All data were checked, corrected and revised using the recorded voices. To examine the transferability of the study, data were made available to some infertile persons who did not participate in the study, asking them to compare the results with their own experiences (23). This study has an ethics code of IR.ACECR.Avicenna.rec.1396.28.

## Results

A total of 40 infertile women and men between the ages of 21–43 were interviewed. Three stages of coding (Open, pivotal, and selective) were done to understand the failure in making identity, as the core category. The participants in interviews were 27 employed and 13 unemployed individuals. Although all men participating in the research were employed, 7 women participating in the research were employed and 13 were housewives. Participants of study were heterogeneous in terms of education and socioeconomic status and belonged to different social and economic classes. In terms of education, 20 subjects had a bachelor’s degree and above, 15 had diploma and 5 had no diploma. 20 participants were living in Tehran and 20 were from other cities (Table 1).

**Table 1.** The characteristics of the participants

**NP**	**Gender**	**Age (Y)**	**Occupation**	**City of residence**	**Education**	**Duration of infertility (Y)**	**Cause of infertility**	**Type of infertility treatment**
**P1**	Female	32	Housewife	Tabriz	High school diploma	12	F ^*^	IVF
**P2**	Female	30	Housewife	Gachsaran	High school diploma	5	F	IVF
**P3**	Female	29	Employee	Tehran	B.Sc	7	F	IVF
**P4**	Female	28	Employee	Tehran	B.Sc	8	F	IVF
**P5**	Female	31	Employee	Tehran	Master’s degree	3	F	IVF
**P6**	Female	28	Employee	Zanjan	High school diploma	10	M ^**^	IUI
**P7**	Female	30	Housewife	Kermanshah	High school diploma	10	F	IVF
**P8**	Female	29	Employee	Tehran	Master’s degree	7	M	IVF
**P9**	Female	35	Housewife	Tabriz	B.Sc	2	F+M	IVF
**P10**	Female	28	Employee	Hamedan	High school diploma	5	M	IUI
**P11**	Female	34	Housewife	Bandar-Abbas	B.Sc	3	F+M	IVF
**P12**	Female	32	Housewife	Shahriyar	Middle school	10	F	IVF
**P13**	Female	42	Housewife	Tehran	B.Sc	3	F	IVF
**P14**	Female	33	Housewife	Tehran	High school diploma	5	F+M	IVF
**P15**	Female	28	Employee	Tehran	B.Sc	4	F	IVF + IUI
**P16**	Female	28	Employee	Tabriz	Master’s degree	5	F	IUI + M
**P17**	Female	30	Housewife	Javanroud	Middle school	3	F	IUI
**P18**	Female	37	Housewife	Tehran	B.Sc	10	F	IVF
**P19**	Female	21	Housewife	Tehran	High school diploma	2	F	IVF
**P20**	Female	32	Housewife	Islam shahr	Middle school	12	F+M	IUI
**P21**	Male	38	Employee	Tehran	High school diploma	5	M	IUI
**P22**	Male	27	Employee	Tehran	Middle school	12	M	IVF
**P23**	Male	37	Employee	Baneh	High school diploma	5	M	IVF
**P24**	Male	34	Employee	Shabestar	High school diploma	7	M	IVF
**P25**	Male	29	Employee	Sannandaj	B.Sc	8	M	IVF
**P26**	Male	36	Employee	Bushehr	B.Sc	3	M	IUI
**P27**	Male	43	Employee	Tehran	High school diploma	10	M	IUI
**P28**	Male	27	Employee	Ahvaz	Middle school	10	F+M	IUI
**P29**	Male	32	Employee	Tehran	B.Sc	7	F+M	IUI
**P30**	Male	34	Employee	Tehran	High school diploma	2	M	Varicocele surgery
**P31**	Male	28	Employee	Tehran	Master’s degree	5	F+M	IVF
**P32**	Male	30	Employee	Bam	B.Sc	3	M	Varicocele surgery
**P33**	Male	28	Employee	Tehran	High school diploma	10	M	IVF
**P34**	Male	33	Employee	Tehran	B.Sc	3	M	IVF
**P35**	Male	30	Employee	Kashan	B.Sc	5	M	IVF
**P36**	Male	36	Employee	Tehran	B.Sc	4	M	IVF
**P37**	Male	32	Employee	Langarud	High school diploma	5	M	IVF
**P38**	Male	35	Employee	Tehran	B.Sc	3	M	IVF
**P39**	Male	31	Employee	Karaj	High school diploma	10	F+M	IVF
**P40**	Male	37	Employee	Tehran	B.Sc	2	M	IVF

* F: Female;

** Male

**Figure 1.**
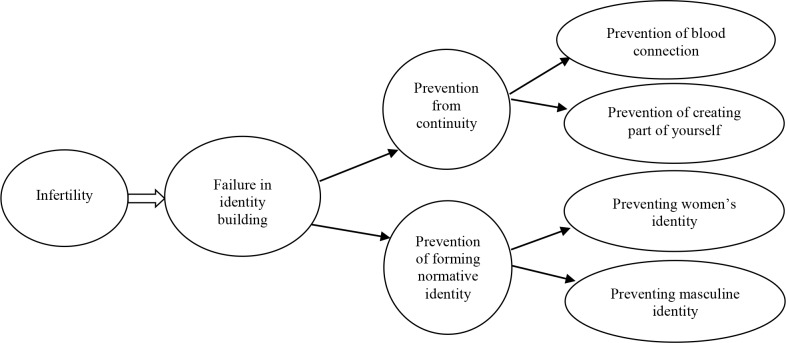
Research paradigm model

The main extracted concepts are included in 2 main categories and sub-categories and concepts by means of Strauss and Corbin coding paradigm are summarized in figure 1. The core category is the chief phenomena around which the sub-categories are built. The aim is to develop core category that explains the problem that the participants are grappling with. Sub-categories define the context.

The core category of infertility is failure in identity building and main categories are prevention from continuity and prevention of forming normative identity and sub-categories are blood connection and creating part of yourself and their common feature is searching yourself; it means that humans tend to continue their generations which explains the main category.

The blood connection refers to the fact that family loyalty is considered to be biologically related. Couples may feel the pressure inside and out of the family to become parents. Parenting is a kind of family development that immediately changes the relationship between the new parent and their parents. The emotional and biological continuity of the generation to another generation forms the family identity and heritage. Therefore, infertile people seek treatment in order to maintain their own continuity through having a biological child with a blood connection who is part of themselves.

In many developing countries, biological motherhood is considered to be the ultimate fate of a woman; most women in this study have expressed “biological motherhood” as their most beautiful and greatest wish, and it is considered as the most important role and responsibility of their life: Motherhood is awesome, every woman wishes to become a mother, when a person gives birth to a child who is part of her body and has a blood connection, she finds meaning in her life and is somehow perfect ... (Interviewee #5).

In the context of the importance of blood connection between the parent and significance of having a biological child who maintains peoples’ continuity, interviewee #12 in an interview with the researcher said:

We can’t even think about adopting a child, because my husband’s family are traditional and a noble family, and their generation is very important for them. They want their family continuity, and they believe in the blood connections. They do not want a child with unclear lineage to come into the family ... . I mean, I know that nobody at all accepts adapting … .

In this regard, men participating in the study also had direct indications of a strong desire to have a biological child and blood connection:

Everyone likes to have a baby. People say that baby is very sweet, although having a child is a heavy responsibility and a lot of expenses and so many problems, but a kid is desired ... Finally, you have to have a result in your life ... my wife also knows how much I do want to have my own baby. As much as I want to hug my baby, I’ll spend money for treatment … .

Women expressed the strong interest in having a biological child while most of them couldn’t reasonably justify their desire to become a mother and simply they referred to motherhood as a natural course and purpose, the philosophy of marriage and act of imitation of others’ behavior. The sense of deprivation of maternal experiences was among the most commonly reported issues of infertile persons:

When you hear other people talking about how good is having a baby, you desire to have your own baby. You want to experience a child’s raising difficulties and maternal feelings like others … .

There is a feeling that everyone likes. I see people around me who talk about their children. I want to experience paternal feelings just like others ... . I always say they are very lucky to have their children’s … .

In the context of the desire of men for generation continuity and creating part of their existence, Jalal al-e-Ahmad in his book “The stone upon a grave” said: “Everyone is a stone upon his father’s grave” and all the subject of the book is to find a person who becomes a stone upon a grave of a man who had never been a father. Jalal al-e-Ahmad describes the autobiography of a man who imposes treatments on his wife who is suffering for many years, although he is the cause of infertility. In fact, in this book, the person’s identity is summarized as “being parent’s continuity” or “affiliated with a child”. Every human being’s need for continuation is inevitable, obvious and instinctive. Jalal writes in his book: “Now you have to stand as the last person in the queue and watch passing of others with regret and the reality is that there is no one from me. It is a road to the edge of the abyss which is then cut. Could you think that you do not have someone behind you to deliver the burden? (19). Interviewee #9 in this regard said:

My husband loves children ... he says I want to have my own child, I do not want my generation to be extinct *… .*

In fact, here, there is the pressure of social expectations and inner social norms on an infertile man, which has cast a shadow over many of his beliefs. Being the only biological child and ending up with a common life and surviving is ignored under the pressure of society and social values. The fact that only having a child is not a sign of the perfection of life and guaranteeing the identity of a man is often ignored under the pressure of society and social values. In the same issue, insisting on the existence of blood ties and the fact that other alternative ways of childbearing, such as adoption are rejected by individuals are common complications; one of the men who participated in this study and was faced with 17 years of infertility (Interviewee #4), said:

I even thought about adopting, but I do not like it at all, I am ready to go for treatment everywhere and spend a lot of time and money, but I prefer to raise my own child ... we either want to have children or stay forever without child. We do not know the lineage of adopted child ..., finally, it is in our destiny to have children or not.

The main category of normative identity consists of the sub-categories of feminine identity (Femininity) and masculine identity (Masculinity), which suggests an attempt to shape a normative identity and the normative social expectation of parenting affiliation with self-perceiving as an ideal woman and man as expected by the community.

The characteristic of this category is the search for ideal identity. Infertility in developing countries is defined as an unfulfilled human identity. In the infertility experience, identity changes occur in individuals and their infertile identity is shaped. The results of the interviews revealed that for those who experience more stress and distress in the infertility process, the infertile identity becomes their central and primary identity. Infertility in women prevents them from fully understanding their feminine identity, and infertile women do not fully identify their femininity from a personal and social dimension. Having a child is essential for the development of gender identity for women and as an adult in many cultures. All the women the researcher spoke to believed that there was no optional alternative for them in the condition of childlessness. In developing societies, in particular, the emphasis is on maternity in shaping women’s identity and having a child may be the key to reaching the status of an adult and receiving social acceptance for women. Infertile woman also has an infertile woman’s identity. She identifies herself as an infertile person, and infertility molds all aspects of their identity:

Infertile woman has a lot of troubles in her life, all of which is for the sake of a child. What can you say if you say something when they ask you: Have you had a baby you expected so much? … finally, I always say to myself that my husband has the right, because all the women I know had given birth to a child, but I could not even do this.

When I found out I cannot be pregnant, I felt so bad. I also have this feeling now. You do not feel happy when you finally realize that you don’t have something that everybody has, you can’t do something that is easy for everyone … you look around, and you see that every woman around you has become mother, and you’re not a mother. It’s a pretty painful sensation for a woman that becomes complete with motherhood.

I used to think that infertility is a kind of flaw and defect, although I am educated and I should be more open minded ... . I always saw it as a subject that I should hide from others.

When an infertile woman encountered with the difference between the identity of an infertile woman and the identity of a mother, emotional turmoil began. Since the infertile woman cannot achieve her ideal, part of her identity will be ruined. But as long as there is hope for her, in addition to her desire for being a mother, she finds it accessible and this impairment of identity does not prevent her from continuing the treatment.

I am returning again after each break because I am hopeful, I will turn to treatment and I say, maybe this time ... . I am very persistent. Finally, new methods have come, every day the scientists are finding new medicines. Infertile people are facing many difficulties but I heard that some of them finally hugged their children.

According to family systems theory, there are a number of plausible stages throughout the life cycle that most people are going through it in a predictable way. Motherhood is the main picture that women become familiar with during their childhood, and motherhood is the mainstay of women’s views. In fact, women always devote a place in their heart for being a mother to find an identity (Interviewee **#**15):

Motherhood is something special and in all women there is a tendency for being a mother. Yesterday, we were our relative’s guest, who had newly bought a doll for her baby. She had played with the doll all the time, gave her bottle and changed her diaper. I think maternal feeling is with us from childhood.

When I see my mother, I would love to be a mother just like her ... . I always liked to make a great family like my mom who made it for me.

On the other hand, in addition to the fact that women become acquainted with the concept of motherhood from childhood, thereby making their identity as a woman, men and women both expect women to be caring and self-sacrificing. As the social construction theory points out, many human categories determined by the physical appearance of individuals, such as men or women, are strongly associated with social assumptions and behaviors. It is assumed that caring for and taking care are the characteristics of women, because only women can be mothers; when they become socialized, driven by the assumption that this role is natural, so they are diverted to caring roles. In women, perception of oneself is deeply influenced by this attitude:

I loved the kids from the beginning, and I always looked after the children in the crowds. I was also a coach for a period ... . I have a great relationship with kids and I really like working with kids.

Women love kids altogether, they like to raise their own children. I’ve been always fond of children and very nice with kids. Everyone tells me that being a mother is very suitable for me.

In interviews with infertile women and men, emotional responses of men to infertility were lower than women’s responses. But the fact is that the feeling after infertility diagnosis for couples is more unpleasant for men as it is reported to be for women. But men’s distress was more linked to the lack of power of masculinity than the absence of a child. This means that although fatherhood is not the center of masculinity, men are not exempted from the effects of culture and society. Masculinity, as imagined by society, is the man’s ability to prove power and merit; however, an infertile man fails to prove this characteristic. In general, infertile men report a lack of identity as a man, a husband and a father and state that they feel they do not have the potential of a man.

If the acquaintances understand the fertility problem, their behavior will change... now we have a person in our family who has a fertility problem ... my brother- in- law has infertility problem, when people find out he will be ridiculed in every party and gathering ..., people even doubt the whole power of his masculinity. He doesn’t go out too much; he stays home and became depressed. I don’t want to be like him, so we did not tell anyone about our problem.

The pressure from the questioning of male masculinity is such that in cases where men’s infertility has been diagnosed, men are still in a state of denial and pessimism towards doctors’ diagnosis. Interviewee #6 said:

I still do not really think that I have fertility problem. There is nothing definitive, I am an athlete, I’m strapped and I believe that I have no problem with fertility ... my wife becomes pregnant easily ... at first, my wife found another center, I said, okay, let’s go. I went there and saw on the bulletin of Infertility Center, “We do not have a problem with fertility” ... I didn’t go inside. Then, she later found here. I found out that it was not just for infertility, and that this place is a frequent abortion center, and that’s why we came.

As long as the true identity of women and men is formed or completed through becoming a parent, not only people with fertility problems and socio-psychological are affected, but they also strongly try to become fertile. With an emphasis on the true social identity and the ideal social identity, a more complete understanding of the effects of infertility can be gained. In interviews with infertile women and men, for professional reasons, they were provided with social work counseling. Many people said they had felt more relaxed and happy and it would be more satisfactory if they could receive counseling services.

## Discussion

The results of the interviews showed that, in general, infertile people (men and women) not only experience failure in child-bearing, but also in many aspects of life, including the purpose of life, the experience of pregnancy, fertility, personal identity, sexual identity, they also experience failure. In the Iranian society, women are often considered to be the cause of infertility, but failing to build identity for both genders exists.

Infertility interfered with the identity of couples in a way that they question their competence, adequacy and ability. Therefore, infertile people may experience identity crisis, because parenting is often interpreted as the gateway to adulthood, and society expects married couples to become parents. The sense of self-worth, sexual identity, and the meaning of marriage are usually confirmed through parenting, and failing to perform parenting role may lead to a major defeat in identity building.

In addition to the struggle to meet social expectations, infertile couples also have to cope with the stigma associated with not having a child. Childlessness is a scandal in many cultures. According to researches, individuals and couples without children often seem immature, selfish, dissatisfied with marriage, career oriented, and psychologically incompatible (24, 25). Diagnosis of infertility in individuals leads a person to identity crisis, as the normative social expectation of parenting has not been achieved. Couples receive the message that they are immature and selfish, and lack the adequacy of adults. In this process, stigma, as a social reaction, causes the destruction and damage of individual’s identity. Stigma management is a general phenomenon, a process that occurs wherever there are norms for desirable identity.

Cultural norms encourage reproduction and child-bearing and celebrate parenthood. Childless couples are receiving defamatory labels, followed by infertility stigma, which can negatively affect the identity of infertile people and their interpersonal relationships.

Based on the experience of infertile people, the failure of creating part of yourself is one of the major failures in building identity and a major challenge. It should be noted that “self” is the intrinsic whole of the individual, and people try to help themselves to continue the generation. Infertility makes this process a failure. Not being able to sustain yourself is also a dimension of failure in building identity and a great challenge. People have a strong need to create their own, which is sustainable throughout life. Hence, they struggle to create and sustain themselves. Forming normative identity as another subcategory of the main category of failure in identity building refers to the fact that the infertile people not only fail in continuity of generation, but they also seek to obtain normative identity in the personal, family, and even wider social circles.

It should be added that the recognition of the psychosocial outcomes of infertility based on the experience of the infertile people in this study comparing to other studies, had some similarities and dissimilarities.

The results of this study showed that infertile individuals found their feminine and male identity defective and disturbing, because the true identity of women and men in our society is becoming complete with parenthood. Indeed, in line with the current study, Harris and Daniluk (2010) found that infertile people are confronted with the problem of obtaining personal identity and gender identity (26), and Haghighatian (2014) also in a quantitative study shows that infertility interferes with the identity of couples in a way that competence, adequacy and ability are under question (27).

Many studies have focused on gender differences and their impact on the outcomes of infertility. In a group of these studies, evidence has shown that women experience more infertility stress than men, and infertility for women is more unpleasant (28–30). But the results of the present study showed that men experienced a lot of cognitive-behavioral and emotional-emotional responses. Many of them reported emotions such as anger, injustice, loss of life control, anxiety, and grief. Thus, it can be claimed that the psychosocial outcomes experienced by Iranian women and men are not significantly different, but only their emotional responses are different. Generally, men struggle to deny their infertility, to escape infertility pain and stigma, and they only seem to be less affected by infertility. Men who participated in the research worried about the question of their masculinity and their male identity.

The component of continuity with the concepts of blood transfusion and part of existence as the main category of failure in identity building was conceptualized and used in this study. Therefore, its continuity category can be considered as a unique part of the experience of Iranian infertile people. According to the experience of infertile people, the impossibility of continuity and creating part of yourself ruins both female and male identity in infertile people.

## Conclusion

The issue of identity is important which gives meaning to lives of individuals in the human society and without the framework for forming identity, the lives of individuals have no meaning and the human effort is always on giving meaning to life and finding its purpose. Not achieving the desired identity of individuals culminates in a kind of confusion and uncertainty. Failure to acquire a person’s identity brings a kind of confusion and uncertainty that provides grounds for psychological and social pathology. Therefore, the main challenge that infertility creates for individuals is the failure to shape the desired and expected identity and lack of meaning in the lives of individuals.

Feeling of frustration and inadequate femininity and masculinity in infertile people can lead to the emergence of many problems that will endanger family life and infertile couples’ relationships. Considering the issues raised and the importance of childbearing in Iranian society, family definition of infertility is an understanding of it as a stressor. These attitudes and the sense of losing identity lead people to treatment.

For generalizing the findings of this study to similar conditions in all Iranian infertile individuals, according to these findings, the following suggestions can be provided. Good practice in infertility clinics encompasses more than medical care. They should pay special attention to the psychological and social aspects of infertility, and through the establishment of social work units in infertility treatment centers, provide various social services and advice to infertile couples. Regarding the prevalence and high rates of infertility in Iranian society, the need for policy making and planning for this target group is important. The results of this research can be in line with the appropriate focus areas for policy and planning to increase the quality of life of infertile people in the community. Infertility in lower socioeconomic groups who can’t provide counseling costs is more prevalent, and the socio-cultural background of each community plays an important role in the consequences of infertility. By establishing a comprehensive health-care approach, in all social care centers, infertility centers are established to enable clients to benefit from the services of a free social worker, along with medical treatment and support.

Findings from grounded theory lead to providing an understanding of the psychosocial outcomes of infertility for those who came to the Avicenna Fertility Center. The theory derived from this section can be a primary resource for infertility researchers in order to provide a better understanding of the psychosocial outcomes of infertility.
